# Factors associated with discharge destination from acute care after moderate-to-severe traumatic injuries in Norway: a prospective population-based study

**DOI:** 10.1186/s40621-023-00431-y

**Published:** 2023-04-13

**Authors:** Håkon Øgreid Moksnes, Christoph Schäfer, Mari Storli Rasmussen, Helene Lundgaard Søberg, Olav Røise, Audny Anke, Cecilie Røe, Pål Aksel Næss, Christine Gaarder, Eirik Helseth, Hilde Margrete Dahl, Morten Hestnes, Cathrine Brunborg, Nada Andelic, Torgeir Hellstrøm

**Affiliations:** 1 Department of Physical Medicine and Rehabilitation, Oslo University Hospital, P.O. Box 4956, Nydalen, 0424 Oslo, Norway; 2 Faculty of Medicine, Research Centre for Habilitation and Rehabilitation Models and Services (CHARM), Institute of Health and Society, University of Oslo, P.O. Box 1072, Blindern, 0316 Oslo, Norway; 3 Department of Clinical Medicine, Faculty of Health Sciences, UiT The Arctic University of Norway, P.O. Box 6050, Langnes, 9037 Tromsø, Norway; 4 Department of Rehabilitation, University Hospital of North Norway, P.O. Box 100, 9038 Tromsø, Norway; 5 Faculty of Health Sciences, Oslo Metropolitan University, P.O. Box 4, St. Olavs Plass, 0130 Oslo, Norway; 6 Norwegian Trauma Registry, Division of Orthopaedic Surgery, Oslo University Hospital, P.O. Box 4956, Nydalen, 0424 Oslo, Norway; 7 Faculty of Medicine, Institute of Clinical Medicine, University of Oslo, P.O. Box 1072, Blindern, 0316 Oslo, Norway; 8 Department of Traumatology, Oslo University Hospital, P.O. Box 4956, Nydalen, 0424 Oslo, Norway; 9 Department of Neurosurgery, Oslo University Hospital, P.O. Box 4956, Nydalen, 0424 Oslo, Norway; 10 Department of Child Neurology, Oslo University Hospital, P.O. Box 4956, Nydalen, 0424 Oslo, Norway; 11 Division of Emergencies and Critical Care, Department of Research and Development, Oslo University Hospital, P.O. Box 4956, Nydalen, 0424 Oslo, Norway; 12 Oslo University Hospital Trauma Registry, Oslo University Hospital, P.O. Box 4956, Nydalen, 0424 Oslo, Norway; 13 Oslo Centre for Biostatistics and Epidemiology, Research Support Services, Oslo University Hospital, P.O. Box 4956, Nydalen, 0424 Oslo, Norway

**Keywords:** Trauma, Trauma center, Traumatic injury, Multiple injury, Discharge destination, Epidemiology

## Abstract

**Background:**

Previous studies have demonstrated that the trauma population has needs for rehabilitation services that are best provided in a continuous and coordinated way. The discharge destination after acute care is the second step to ensuring quality of care. There is a lack of knowledge regarding the factors associated with the discharge destination for the overall trauma population. This paper aims to identify sociodemographic, geographical, and injury-related factors associated with discharge destination following acute care at trauma centers for patients with moderate-to-severe traumatic injuries.

**Methods:**

A multicenter, population-based, prospective study was conducted with patients of all ages with traumatic injury [New Injury Severity Score (NISS) > 9] admitted within 72 h after the injury to regional trauma centers in southeastern and northern Norway over a 1-year period (2020).

**Results:**

In total, 601 patients were included; a majority (76%) sustained severe injuries, and 22% were discharged directly to specialized rehabilitation. Children were primarily discharged home, and most of the patients ≥ 65 years to their local hospital. Depending on the centrality of their residence [Norwegian Centrality Index (NCI) 1–6, where 1 is most central], we found that patients residing in NCI 3–4 and 5–6 areas sustained more severe injuries than patients residing in NCI 1–2 areas. An increase in the NISS, number of injuries, or a spinal injury with an Abbreviated Injury Scale (AIS) ≥ 3 was associated with discharge to local hospitals and specialized rehabilitation than to home. Patients with an AIS ≥ 3 head injury (RRR 6.1, 95% Confidence interval 2.80–13.38) were significantly more likely to be discharged to specialized rehabilitation than patients with a less severe head injury. Age < 18 years was negatively associated with discharge to a local hospital, while NCI 3–4, preinjury comorbidity, and increased severity of injuries in the lower extremities were positively associated.

**Conclusions:**

Two-thirds of the patients sustained severe traumatic injury, and 22% were discharged directly to specialized rehabilitation. Age, centrality of the residence, preinjury comorbidity, injury severity, length of hospital stay, and the number and specific types of injuries were factors that had the greatest influence on discharge destination.

## Background

Traumatic injuries are a leading global cause of disability in all age groups (WHO 2014; Haagsma et al. 2016). Improvements in acute trauma care have improved survival rates and functional outcomes following severe injury (Gabbe et al. 2012; Mackenzie et al. 2008; Ursic et al. 2008). However, most patients who suffer major trauma still experience poor long-term physical and mental health outcomes and reduced quality of life (Gabbe et al. 2017; Kaske et al. 2014; Havermans et al. 2020; Lyons et al. 2011; Soberg et al. 2012). A study on the lasting impact of trauma found that patients with moderate and severe injuries more often were unable to return to work and were still receiving some form of medical benefit up to 5 years post-injury compared to their pre-injury requirements (Uleberg et al. 2019).

Children constitute a special subgroup of the trauma population, and for children surviving injuries, impairments and the need for care and rehabilitation can have a major impact on the prospects for health, education, and social inclusion (WHO 2008). At the other end of the age spectrum are the geriatric trauma patients, a rapidly growing group in several parts of the world who are at high risk for poor outcomes (Chatterji et al. 2015; Beck et al. 2018). Several studies reported poorer outcomes with increasing age (Gabbe et al. 2016; Holtslag et al. 2006; Polinder et al. 2007), and elderly patients are at increased risk of morbidity after injury in both inpatient and the post-discharge settings (Strosberg et al. 2016).

Both treatment and rehabilitation in the acute phase are important to minimize the patients’ impairments and to attain favorable patient outcomes. Coordinated and continuous multidisciplinary rehabilitation and health services are also recommended in the post-acute phase (Turner-Stokes et al. 2015). However, a systematic understanding of both the rehabilitation needs and the existing rehabilitation processes for the trauma population at large is lacking. Both study based on quasi-experimental design and limited age-range and the prospective follow-up study have shown improved outcome for patients with severe traumatic brain injury (TBI) who receive early rehabilitation in the ICU unit and follow a direct pathway to inpatient rehabilitation (Andelic et al. 2012; Sveen et al. 2016; Anke et al. 2015). Furthermore, there is a lack of knowledge on how geographical factors influence rehabilitation services and patient transfer between levels (Jeppesen et al. 2020), and there is a clear need to develop a larger evidence base on regional variation in recovery following injury to inform the optimization of post-discharge care services (Keeves et al. 2019). Increased knowledge about how sociodemographic and injury-related factors influence the clinician’s choice of discharge destination is needed. A previous study found that having a TBI, spinal cord injury, injuries of the pelvis or lower extremities, increased age, prolonged length of stay (LOS) in the intensive care unit (ICU), suicide attempt, or intubation in the ICU were associated with an increased likelihood of being transferred to a rehabilitation clinic following trauma (Debus et al. 2016). A study on patients with TBI found that comorbidity, LOS, and number of days in the ICU were factors that significantly influenced the discharge destination (Chen et al. 2012). They also highlighted rural location as a potential influencing factor on discharge destination. However, there is inconsistency in the findings among studies reporting on the relationship between geographical location and in-hospital outcomes after injury (Keeves et al. 2019).

Therefore, to guide the improvement of rehabilitation planning, there is a need for studies that include trauma patients of all ages, assess discharge destinations, and compare post-injury functioning, rehabilitation needs, and processes.

The primary aim of this population-based study was to identify sociodemographic and injury-related factors associated with discharge destination following acute care at the trauma center for patients with moderate-to-severe injuries. The secondary aim was to further describe the patients’ epidemiological characteristics, including geographical differences based on the Norwegian Centrality Index (NCI). We hypothesized that a higher proportion of patients with severe injuries would be discharged to specialized rehabilitation than patients with moderate injuries, independent of the geographical location of their residence.

## Methods

### Setting and participants

A multicenter cohort study was conducted using prospectively collected data from patients admitted to the regional trauma centers at Oslo University Hospital (OUH) and the University Hospital of North Norway (UNN) and who were followed at 6 and 12 months post-injury. OUH serves as the regional trauma center for the southeast of Norway (population approximately 3.0 million), and UNN serves as the regional trauma center for the north of Norway (population approximately 482,000 (Statistics Norway 2022)). Both hospitals also serve as local trauma hospitals; UNN for Tromsø and surroundings (population approximately 193,000) and OUH for Oslo (population approximately 700,000). In Norway, the healthcare system is publicly funded, and aims to provide universally accessible healthcare. This includes hospital-based specialist care and outpatient clinics, rehabilitation services and community-based care.

### Inclusion and exclusion criteria

Patients of all ages with a New Injury Severity Score (NISS) > 9 (using the 2008 update of the 2005 Abbreviated Injury Scale [AIS] (2005) who were admitted over a 1-year period (2020) and discharged alive were assessed for eligibility. The recruitment period at OUH was from 01.01.2020 to 31.12.2020, and the recruitment period at UNN was from 01.02.2020 to 31.01.2021. Other inclusion criteria were Norwegian residents admitted directly or after transfer from local hospitals within 72 h of injury and with at least a two-day hospital stay. The inclusion criterion of NISS > 9 is based on guidance from the National Institute for Health and Care Excellence, which recommends that patients with an Injury Severity Score (ISS) > 9 in a trauma unit to be assessed for rehabilitation needs and rehabilitation prescriptions (NICE guideline [NG40] 2016). The exclusion criteria were non-Norwegian residents and non-Norwegian or non-English speakers. The study protocol was published in 2021 (Soberg et al. 2021).

### Procedures

Patients were identified by one of the physicians allocated to the project through participation in the daily trauma report meetings, from lists of new hospitalized patients registered by the Trauma Department, and by searches performed on the hospital administrative medical record system using an admission diagnosis of trauma. Once it was confirmed that a patient fulfilled the inclusion criteria, the research assistant or the study physician provided study information to the patient, caregiver, and/or parents in case of children.

AIS-certified physicians (H.M., C.S., O.R., R.B., and N.A.) registered injury severity at inclusion. These scores were validated against the scores recorded in the hospital-based trauma registries, and the registries’ scores were used in the study.

### Variables, definitions, and data collection

Trauma- and treatment-related data, mechanism of injury, data on work-related injury, non-surgical and surgical procedures, LOS and discharge destination were obtained from the medical records. Injury-related data (i.e., body regions affected, number of injuries, and AIS/NISS) were collected from the hospital-based trauma registries. LOS was defined as days in ICU and the surgical departments. Sociodemographic data, including age, sex, marital status, formal education, municipality of residence, and preinjury comorbidity status were obtained from the medical records and from patients or caregiver.

Outcomes: The main outcome in the current study was discharge destination from the acute care units at the trauma centers (OUH and UNN) and was categorized as: (1) home, (2) local hospital, or (3) specialized rehabilitation. The acute care units comprised the ICU and the surgical departments. “Home” was defined as a home residence with or without support. Only 19 patients (3.2% of the total study cohort) were discharged to nursing homes or community-based inpatient rehabilitation and were included in the “local hospital” group. “Specialized rehabilitation” was defined as rehabilitation in a hospital or institution that is a part of the specialist health care system.

Age was categorized into the following eight groups: 0–17, 18–24, 25–34, 35–44, 45–54, 55–64, 65–74, and ≥ 75 years. In the multinomial logistic regression/statistical analyses, we pooled age into four categories: 0–17, 18–34, 35–64, and ≥ 65 years.

Marital status was categorized into three groups: “Married/cohabitant”, “lives with parents” (children) and “single/lives alone”.

Pre-injury comorbidity was measured in two ways: using the American Society of Anesthesiologists physical status (ASA-PS) classification system (2014); this was the variable used in the regression analysis. The assigned ASA increases with more comorbidities (i.e., a normal, healthy patient is assigned ASA 1). No patients were assigned ASA 5 or 6, so these groups are not presented in the results. There was only one patient assigned ASA 4, so groups 3 and 4 were merged. Furthermore, comorbidity was categorized into preinjury mental health or drug/alcohol condition (defined as a comorbidity if information about the condition was found in the medical record), neurological, muscular/skeletal, cardiac/vascular, “other,” and several (if more than one condition in one or more of the groups mentioned). This variable was also dichotomized for the analysis of differences, with NCI as the dependent variable.

We categorized education into three groups for Table 1: low, primary school/high school (0–12/13 years); high, university (13/14–16/ ≥ 17 years); and children (< 18 years of age). For Table 2 we categorized education level into five groups for the analysis of sociodemographic data versus NCI: primary school (0–9/10 years), high school (10/11–12/13 years), university (13/14–16/17 years), university (> 16/17 years), and children (< 18 years of age). Children were categorized into their own group as a substantial proportion had not completed their education.

**Table 1:** Sociodemographic and injury-related characteristics according to discharge destination

Characteristic	Overall *n* (Col%)	Home n (Col%, Row%)	Local hospital *n* (Col%, Row%)	Specialized rehabilitation *n* (Col%, Row%)	*p*-value
Total	601 (100)	211 (100, 35.1)	256 (100, 42.6)	134 (100, 22.3)	
Sex					
Male	451 (75.0)	162 (76.8, 35.9)	188 (73.4, 41.7)	101 (75.4, 22.4)	0.705
Female	150 (25.0)	49 (23.2, 32.7)	68 (26.6, 45.3)	33 (24.6, 22.0)	
Age (years)					
< 18	63 (10.5)	34 (16.1, 54.0)	12 (4.7,19.0)	17 (12.7, 27.0)	** < 0.001**
18–24	48 (8.0)	23 (10.9, 47.9)	11 (4.3, 22.9)	14 (10.4, 29.2)	
25–34	75 (12.5)	30 (14.2, 40.0)	27 (10.5, 36.0)	18 (13.4, 24.0)	
35–44	75 (12.5)	28 (13.3, 37.3)	31 (12.1, 41.3)	16(11.9, 21.3)	
45–54	86 (14.3)	35 (16.6, 40.7)	34 (13.3, 39.5)	17 (12.7, 19.8)	
55–64	103 (17.1)	30 (14.2, 29.1)	46 (18.0, 44.7)	27 (20.1, 26.2)	
65–74	101 (16.8)	19 (9.0, 18.8)	62 (24.2, 61.4)	20 (14.9, 19.8)	
≥ 75	50 (8.3)	12 (5.7, 24.0)	33 (12.9, 66.0)	5 (3.7, 10.0)	
Marital status^a^					
Married/cohabitant	309 (51.6)	93 (44.3, 30.1)	150 (58.8, 48.5)	66 (49.3, 21.4)	** < 0.001**
Lives with parents	75 (12.5)	39 (18.6, 52.0)	16 (6.3, 21.3)	20 (14.9, 26.7)	
Single/lives alone	215 (35.9)	78 (37.1, 36.3)	89 (34.9, 41.4)	48 (35.8, 22.3)	
Level of education^b^					
Low (Primary school/High school [0–12/13 years])	287 (51)	81 (40.1, 28.2)	142 (60.4, 49.5)	64 (50.8, 22.3)	** < 0.001**
High (University [13/14–16/ ≥ 17 years])	214 (38)	87 (43.1, 40.7)	81 (34.5, 37.9)	46 (36.5, 21.5)	
Children (< 18 years)	62 (11.0)	34 (16.8, 54.8)	12 (5.1, 19.4)	16 (12.7, 25.8)	
Centrality Index (NCI)					
Category 1 & 2	337 (56.1)	140 (66.4, 41.5)	123 (48.0, 36.5)	74 (55.2, 22.0)	** < 0.001**
Category 3 & 4	201 (33.4)	48 (22.7, 23.9)	109 (42.6, 54.2)	44 (32.8, 21.9)	
Category 5 & 6	63 (10.5)	23 (10.9, 36.5)	24 (9.4, 38.1)	16 (11.9, 25.4)	
Pre-injury comorbidity					
ASA 1	327 (54.4)	143 (67.8, 43.7)	105 (41.0, 32.1)	79 (59.0, 24.2)	** < 0.001**
ASA 2	205 (34.1)	49 (23.2, 23.9)	110 (43.0, 53.7)	46 (34.3, 22.4)	
ASA 3 & 4	69 (11.5)	19 (9.0, 27.5)	41 (16.0, 59.4)	9 (6.7, 13.0)	
Preinjury comorbidity status					
Mental health or drug/alcohol condition	133 (22.1)	29 (13.7, 21.8)	75 (29.3, 56.4)	29 (21.6, 21.8)	** < 0.001**
Neurological	58 (9.7)	15 (7.1, 25.9)	30 (11.7, 51.7)	13 (9.7, 22.4)	0.356
Muscular/skeletal	99 (16.5)	24 (11.4, 24.2)	48 (18.8, 48.5)	27 (20.1, 27.3)	0.115
Cardiac/vascular	140 (23.3)	38 (18.0, 27.1)	72 (28.1, 51.4)	30 (22.4, 21.4)	0.071
Other	187 (31.1)	48 (22.7, 25.7)	101 (39.5, 54,0)	38 (28.4, 20.3)	**0.002**
Several	221 (36.8)	52 (24.6, 23.5)	124 (48.4, 56.1)	45 (33.6, 20.4)	** < 0.001**
Injury mechanism					
Falls	243 (40.4)	70 (33.2, 28.8)	113 (44.1, 46.5)	60 (44.8, 24.7)	**0.01**
Transport-related	227 (37.8)	84 (39.8, 37.0)	92 (35.9, 40.5)	51 (38.1, 22.5)	
Violence	18 (3.0)	12 (5.7, 66.7)	2 (0.8, 11.1)	4 (3.0, 22.2)	
Others	113 (18.8)	45 (21.3, 39.8)	49 (19.1, 43.4)	19 (14.2, 16.8)	
Work-related injury^c^					
Yes	42 (7.0)	11 (5.2, 26.2)	20 (7.9, 47.6)	11 (8.2, 26.2)	0.442
No	557 (93.0)	200 (94.8, 35.9)	234 (92.1, 42.0)	123 (91.8, 22.1)	
Injury severity (NISS)					
Median (IQR)	22 (16, 29)	17 (12, 22)	22 (17, 29)	27 (22, 43)	** < 0.001**
Moderate injury (NISS 10–15)	144 (24.0)	86 (40.8, 59.7)	46 (18.0, 31.9)	12 (9.0, 8.3)	** < 0.001**
Severe injury (NISS > 15)	457 (76.0)	125 (59.2, 27.4)	210 (82.0, 46.0)	122 (91.0, 26.7)	
Number of injuries					
1–3	163 (27.1)	89 (42.2, 54.6)	48 (18.8, 29.4)	26 (19.4, 16.0)	** < 0.001**
4–6	227 (37.8)	94 (44.5, 41.4)	94 (36.7, 41.4)	39 (29.1, 17.2)	
> 6	211 (35.1)	28 (13.3, 13.3)	114 (44.5, 54.0)	69 (51.5,32.7)	
Injured body region					
Head	276 (45.9)	65 (30.8, 23.6)	113 (44.1, 40.9)	98 (73.1, 35.5)	** < 0.001**
Face	204 (33.9)	59 (28.0, 28.9)	84 (32.8, 41.2)	61 (45.5, 29.9)	**0.003**
Neck	24 (4.0)	5 (2.4, 20.8)	12 (4.7, 50.0)	7 (5.2, 29.2)	0.316
Thorax	236 (39.3)	78 (37.0, 33.1)	122 (47.7, 51.7)	36 (26.9, 15.3)	** < 0.001**
Abdomen	100 (16.6)	57 (27.0, 57.0)	31 (12.1, 31.0)	12 (9.0, 12.0)	** < 0.001**
Spine	192 (31.9)	39 (18.5, 20.3)	114 (44.5, 59.4)	39 (29.1, 20.3)	** < 0.001**
Upper extremity	184 (30.6)	52 (24.6, 28.3)	102 (39.8, 55.4)	30 (22.4, 16.3)	** < 0.001**
Lower extremity	173 (28.8)	44 (20.9, 25.4)	106 (41.4, 61.3)	23 (17.2, 13.3)	** < 0.001**
External and others	79 (13.1)	25 (11.8, 31.6)	38 (14.8, 48.1)	16 (11.9, 20.3)	0.569
Body region with AIS ≥ 3					
Head	250 (41.6)	53 (25.1, 21.2)	96 (37.5, 38.4)	101 (75.4, 40.4)	** < 0.001**
Face	15 (2.5)	3 (1.4, 20.0)	9 (3.5, 60.0)	3 (2.2, 20.0)	0.417
Neck	11 (1.8)	2 (0.9, 18.2)	4 (1.6, 36.4)	5 (3.7, 45.5)	0.219
Thorax	197 (32.8)	66 (31.3, 33.5)	103 (40.2, 52.3)	28 (20.9, 14.2)	** < 0.001**
Abdomen	73 (12.1)	50 (23.7, 68.5)	16 (6.3, 21.9)	7 (5.2, 9.6)	** < 0.001**
Spine	80 (13.3)	13 (6.2, 16.3)	43 (16.8, 53.8)	24 (17.9, 30.0)	** < 0.001**
Upper extremity	9 (1.5)	4 (1.9, 44.4)	3 (1.2, 33.3)	2 (1.5, 22.2)	0.909
Lower extremity	92 (15.3)	18 (8.5, 19.6)	62 (24.2, 67.4)	12 (9.0, 13.0)	** < 0.001**
External and others	2 (0.3)	0 (0, 0)	2 (0.8, 100)	0 (0, 0)	0.51
Surgical procedures					
No	214 (35.6)	98 (46.4, 45.8)	77 (30.1, 36.0)	39 (29.1, 18.2)	** < 0.001**
Yes	387 (64.4)	113 (53.6, 29.2)	179 (69.9, 46.3)	95 (70.9, 24.5)	
Non-surgical procedures					
No	94 (15.6)	63 (29.9, 67.0)	29 (11.3, 30.9)	2 (1.5, 2.1)	** < 0.001**
Yes	507 (84.4)	148 (70.1, 29.2)	227 (88.7, 44.8)	132 (98.5, 26.0)	
Length of hospital stay (acute care unit at the trauma center), days					
Median (IQR)	6 (3, 10)	5 (3, 8)	5 (3, 9.75)	10 (4, 17)	** < 0.001**

For each independent variable, the corresponding number of patients discharged to the different destinations is presented together with the proportion this number constitutes of the column total (Col%) and of the row total (Row%). Local hospital (n = 256) consists of local hospital (n = 237, 92.6%) and nursing home/inpatient community-based rehabilitation (n = 19, 7.4%)

Significant results are presented in bold

*ASA* American Society of Anesthesiologists classification, *NISS* New Injury Severity Score, *AIS* Abbreviated Injury Scale, *LOS* length of hospital stay, *IQR* interquartile range

^a^Missing = 2, ^b^Missing = 38, ^c^Missing = 2

**Table 2:** Baseline patient characteristics and differences by NCI (Norwegian Centrality Index)

Characteristic	Overall *n* (Col %)	Centrality index (NCI) 1–2 *n* (Col %)	Centrality index (NCI) 3–4 *n* (Col %)	Centrality index (NCI) 5–6 *n* (Col %)	*p*-value
Total	601	337	201	63	
Age (years), mean (SD)	46.88 (21.2)	47.63 (20.4)	45.42 (21.7)	47.54 (23.8)	0.49
Sex					
Male	451 (75.0)	247 (73.3)	154 (76.6)	50 (79.4)	0.486
Female	150 (25.0)	90 (26.6)	47 (23.4)	13 (20.6)	
Education^a^					
Primary school (0–9/10 years)	58 (10.3)	26 (8.3)	23 (12.0)	9 (15.3)	** < 0.001**
High School (10/11–12/13 years)	229 (40.7)	109 (34.8)	91 (47.6)	29 (49.2)	
University (13/14–16/17 years)	163 (29.0)	115 (36.7)	39 (20.4)	9 (15.3)	
University (> 16/ > 17 years)	51 (9.1)	39 (12.5)	9 (4.7)	3 (5.1)	
Children (< 18 years)	62 (11.0)	24 (7.7)	29 (15.2)	9 (15.3)	
Preinjury comorbidity					
No comorbidity	221 (36.8)	118 (35.0)	79 (39.3)	24 (38.1)	0.592
≥ 1 comorbidity	380 (63.2)	219 (65.0)	122 (60.7)	39 (61.9)	
Preinjury comorbidity					
Mental health or drug/alcohol condition	133 (22.1)	79 (23.4)	48 (23.9)	6 (9.5)	**0.039**
Neurological	58 (9.7)	32 (9.5)	23 (11.4)	3 (4.8)	0.29
Muscular/skeletal	99 (16.5)	57 (16.9)	30 (14.9)	12 (19.0)	0.704
Cardiac/vascular	140 (23.3)	76 (22.6)	42 (20.9)	22 (34.9)	0.063
Other	187 (31.1)	105 (31.2)	63 (31.3)	19 (30.2)	0.984
Several	221 (36.8)	121 (35.9)	77 (38.3)	23 (36.5)	0.854
Mechanism of injury					
Falls	243 (40.4)	136 (40.4)	90 (44.8)	17 (27.0)	**0.035**
Transport- related injuries	227 (37.8)	133 (39.5)	67 (33.3)	27 (42.9)	
Violence	18 (3.0)	13 (3.9)	5 (2.5)	0 (0.0)	
Other	113 (18.8)	55 (16.3)	39 (19.4)	19 (30.2)	
Injury severity					
Moderate injury (NISS 10–15)	144 (24.0)	97 (28.8)	32 (15.9)	15 (23.8)	**0.003**
Severe injury (NISS > 15)	457 (76.0)	240 (71.2)	169 (84.1)	48 (76.2)	
Injured body region (AIS ≥ 3)					
Head	250 (41.6)	138 (40.9)	91 (45.3)	21 (33.3)	0.229
Face	15 (2.5)	7 (2.1)	7 (3.5)	1 (1.6)	0.63
Neck	11 (1.8)	3 (0.9)	5 (2.5)	3 (4.8)	0.053
Thorax	197 (32.8)	106 (31.5)	68 (33.8)	23 (36.5)	0.681
Abdomen	73 (12.1)	29 (8.6)	35 (17.4)	9 (14.3)	**0.009**
Spine	80 (13.3)	36 (10.7)	29 (14.4)	15 (23.8)	**0.016**
Upper extremity	9 (1.5)	3 (0.9)	4 (2.0)	2 (3.2)	0.22
Lower extremity	92 (15.3)	52 (15.4)	34 (16.9)	6 (9.5)	0.362
External and other	2 (0.4)	2 (0.6)	0 (0.0)	0 (0.0)	0.624
Surgery					
No	214 (35.6)	130 (38.6)	67 (33.3)	17 (27.0)	0.15
Yes	387 (64.4)	207 (61.4)	134 (66.7)	46 (73.0)	
LOS (acute care unit at the trauma center) in days, median (IQR)	6 (3, 10)	5 (3, 9)	6 (4, 11)	8 (5, 13)	** < 0.001**
Discharge destination					
Home	211 (35.1)	140 (41.5)	48 (23.9)	23 (36.5)	** < 0.001**
Local hospital	256 (42.6)	123 (36.5)	109 (54.2)	24 (38.1)	
Specialized rehabilitation	134 (22.3)	74 (22.0)	44 (21.9)	16 (25.4)	

Significant results are presented in bold

*SD* standard deviation, *NISS* New Injury Severity Score, *AIS* Abbreviated Injury Scale, *LOS* length of hospital stay, *IQR* interquartile range

^a^Missing = 38. Local hospital (*n* = 256) consists of local hospitals (*n* = 237, 92.6%) and nursing home/inpatient community-based rehabilitation (*n* = 19, 7.4%)

Any type of performed surgical- and non-surgical procedure was dichotomized into no/yes.

LOS was recorded as number of days in the acute care unit.

Geographical location: We used the NCI as the variable for centrality. The NCI was developed by Statistics Norway as a measure of how centrally municipalities are located in terms of service functions and workplaces that are accessible for a resident within 90 min (Høydahl 2020). The NCI ranges from 1 to 6, where index 1 and 2 denote the most central areas and index 5 and 6 denote the least central areas (Sentralitetsindeksen 2020). The NCI category was determined based on the patient’s municipality of residence. For analysis, the six categories were collapsed into three groups: 1 and 2 (most central, referred to as NCI 1–2), 3 and 4 (referred to as NCI 3–4), and 5 and 6 (least central, referred to as NCI 5–6).

Mechanism of injury was categorized as fall, transport-related injury, violence, or other. The severity of the injury was registered using the NISS. The NISS and the ISS are derived from AIS where each injury is ascribed a body region and a severity code (Association for the Advancement of Automotive Medicine 2005). The NISS incorporates the three most severe injuries regardless of the body regions, while the ISS is calculated by taking the highest AIS severity code in each of the three most severely injured ISS body regions (Lavoie et al. 2005). This means that the NISS and the ISS can differ for the individual patient, where the NISS will have the same or a higher value than the ISS. We chose to use the NISS, as this is the scoring system used by the hospitals’ trauma registries in Norway. It is generally recognized that severe injury is defined as an ISS greater than 15 (Palmer et al. 2016). In this study, we defined a NISS score 10–15 as “moderate”, 16–25 as “severe,” and 26–75 as “profound.” For our analyses, we combined severe and profound into a category named “severe,” which comprised the scores 16–75.

### Statistical analysis

The descriptive characteristics of the study population are reported as frequencies and percentages for categorical data and medians with interquartile ranges (IQRs) or mean with standard deviation (SD) for continuous data.

For comparisons between categories of dependent variables in Tables 1 and 2, the one-way analysis of variance (ANOVA) or Kruskal–Wallis test were used for continuous variables, and the chi-square test or Fisher’s exact test were used for categorical variables, as appropriate. Univariable and multivariable multinomial logistic regression analyses were performed to investigate factors associated with discharge to either specialized rehabilitation or a local hospital compared to discharge to the home. Variables were included in the multivariable models based on knowledge from the literature and expert opinion. The following factors were included: sex, age at time of injury, NCI, preinjury ASA, injury mechanism, NISS, number of injuries, body regions with AIS ≥ 3, and LOS. We present the full multivariable model to show which factors were most strongly associated with discharge destination when taken together, without subsequent elimination of variables driven by our data. We merged the data from the two centers and first performed the whole cohort analysis. Secondly, we performed sensitivity analyses with adjustment for the trauma center the patients belonged to, see Table 5 in the Appendix.

The results are presented as relative risk ratios (RRRs) with a 95% confidence interval (CI). The possible multi-collinearity of the factors was explored using Spearman’s correlation coefficient ≥ 0.7 as a cut-off. McFadden’s *r*^2^ was used as goodness of fit measure with values ranging from 0 to 1, where higher values indicate better model fit. We performed dropout analysis according to differences in sex and age; here, we used Student’s *t*-test and the chi-square test, as appropriate.

All tests were two-sided, and a 5% significance level was used. For the statistical analysis, we used SPSS statistics version 28 (IBM Corp., Armonk, NY, USA), except for the multinomial logistic regression, which was performed using STATA version 17 (Stata Corp LLC, College Station, TX, USA).

## Result

### Participants

**Fig. 1:**
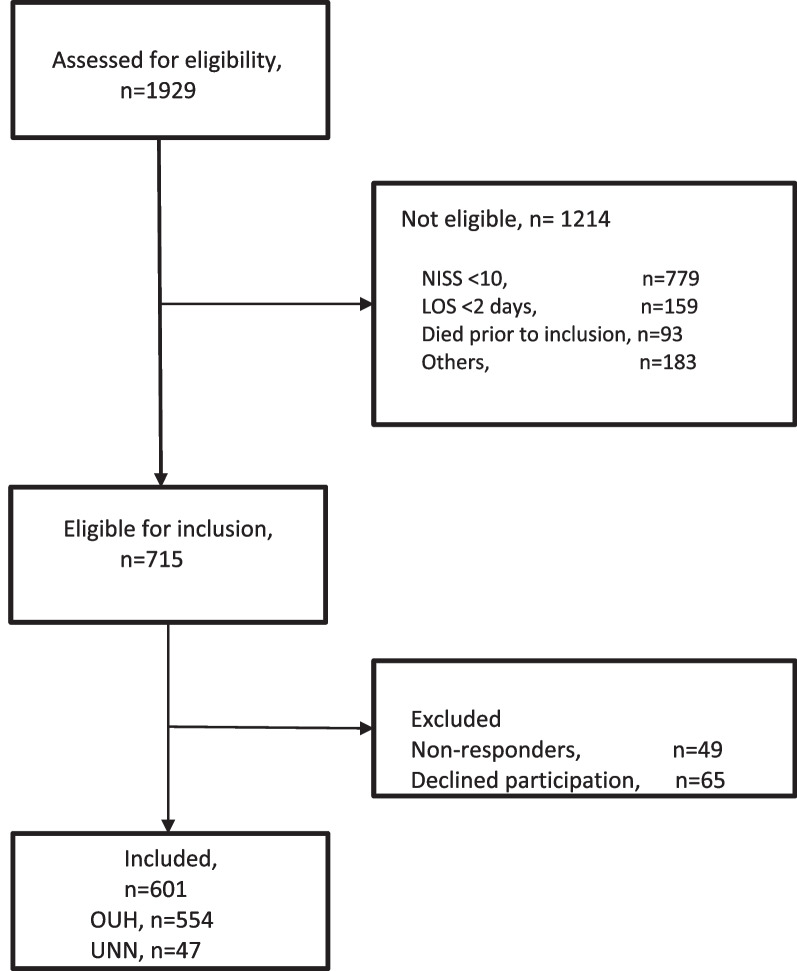
Flowchart. NISS, New Injury Severity Score; LOS, length of hospital stay; OUH, Oslo University Hospital; UNN, University Hospital of North Norway

In total, 1929 patients were assessed for eligibility (Fig. 1). A total of 1214 patients were determined to be ineligible, most frequently due to a NISS < 10 or a LOS less than two days. In total, 601 of the 715 eligible patients were successfully contacted and consented to participate; of these, 47 patients were recruited from UNN and 554 from OUH.

### Patient characteristics

Tables 1 presents patient characteristics by discharge destination.

The mean age of the patients was 47 years (SD: 21.2), and 75% were male. Dropout analysis revealed that the mean age in the dropout group (*n* = 114) was somewhat higher (51 years [SD: 23.1]), while the proportion of males was slightly lower (74%). We found that 35% of patients were discharged to their homes, while the largest group, constituting 43% of the patients, was discharged to a local hospital. A total of 22% were discharged to specialized rehabilitation; for children (< 18 years old), this number was 27%. Most children were discharged home (54%), whereas the local hospital was the most frequent discharge destination for patients ≥ 65 years of age (61% for patients 65–74 years and 66% for patients ≥ 75 years). The proportion of patients discharged to specialized rehabilitation decreased with increasing ASA score, that is, with more co-morbidities. Most of the patients (56%) were residents of the most central municipalities (NCI 1–2). NCI 3–4 was assigned to 33% of the patients and NCI 5–6 to 11%.

Table 2 presents the baseline patient characteristics by NCI. Patients assigned NCI 1–2 had a significantly higher education level than other patients. The frequency of mental health/drug conditions was lowest for patients assigned NCI 5–6. Falls and transport-related events were the dominating causes of injury, with falls being a less frequent mechanism of injury for patients assigned NCI 5–6. Patients assigned NCI 3–4 had a higher proportion of severe injuries, and patients assigned NCI 1–2 had a lower proportion of abdominal injuries with AIS ≥ 3. The frequency of severe spinal injury was higher for patients assigned NCI 5–6. The analysis also revealed that LOS increased with higher NCI. In this unadjusted analysis, discharge to a local hospital was associated with NCI 3–4, and discharge to specialized rehabilitation was associated with NCI 5–6.

### Predisposing factors for discharge destination

The results of the univariable multinomial logistic regression analysis are shown in Table 3. There was a significantly lower RRR for discharge to a local hospital than home for children and young adults and a higher RRR for patients ≥ 65 years compared with the 35–64-years reference age group. Compared with patients assigned NCI 1–2, patients assigned NCI 3–4 had a significantly higher RRR for discharge to specialized rehabilitation (RRR 1.73, 95% CI 1.06–2.85) and a local hospital (RRR 2.58, 95% CI 1.70–3.92) than home. Patients with higher ASA scores had a higher RRR for discharge to a local hospital versus home, and patients with ASA 2 had significantly higher RRR for discharge to specialized rehabilitation compared with patients in the ASA 1 group. Severe head and spinal injuries (AIS ≥ 3) resulted in a higher RRR for discharge to specialized rehabilitation (head: RRR 9.12, 95% CI 5.53–15.06 and spine: RRR 3.32, 95% CI 1.63–6.79). Patients with injuries of the head, spine, thorax, and lower extremities had significantly higher RRR for discharge to a local hospital than home. Abdominal and thorax injuries with AIS ≥ 3 led to a significantly lower RRR for discharge to specialized rehabilitation, and patients with abdominal injuries AIS ≥ 3 also had a lower RRR for discharge to a local hospital than home.

**Table 3:** Univariable multinomial logistic regression for sociodemographic and injury-related characteristics

Characteristic	Univariable RRRs (95% CI)	
*n* = 601	Local hospital versus Home	Specialized rehabilitation versus Home
Sociodemographic characteristics		
Sex		
Male	1	1
Female	1.20 (0.78–1.82)	1.08 (0.65–1.79)
Age at time of injury (years)		
0–17	**0.30 (0.14–0.60)**	0.78 (0.40–1.51)
18–34	**0.60 (0.36–0.99)**	0.94 (0.54–1.62)
35–64	1	1
≥ 65	**2.57(1.57–4.19)**	1.25 (0.67–2.32)
Centrality Index (NCI)		
Category 1 and 2	1	1
Category 3 and 4	**2.58 (1.70–3.92)**	**1.73 (1.06–2.85)**
Category 5 and 6	1.19 (0.64–2.21)	1.32 (0.66–2.64)
Preinjury ASA		
1	1	1
2	**3.06 (2.01–4.66)**	**1.70 (1.04–2.77)**
3 and 4	**2.94 (1.61–5.35)**	0.76 (0.32–1.82)
Injury- and treatment-related characteristics		
Injury mechanism		
Falls	1.42 (0.94–2.14)	1.34 (0.83–2.18)
Transport-related	1	1
Violence	**0.15 (0.03–0.70)**	0.55 (0.17–1.79)
Others	1.03 (0.61–1.74)	0.72 (0.34–1.40)
NISS		
Moderate (NISS 10–15)	1	1
Severe (NISS > 15)	**3.14 (2.06–4.78)**	**6.99 (3.64–13.45)**
Number of injuries	**1.30 (1.21–1.40)**	**1.33 (1.23–1.44)**
Body regions with AIS ≥ 3		
Head	**1.79 (1.20–2.67)**	**9.12 (5.53–15.06)**
Face	2.53 (0.68–9.45)	1.59 (0.32–7.98)
Neck	1.66 (0.30–9.15)	4.05 (0.77–21.18)
Thorax	**1.48 (1.01–2.17)**	**0.58 (0.35–0.96)**
Abdomen	**0.21 (0.12–0.39)**	**0.18 (0.08–0.40)**
Spine	**3.07 (1.61–5.89)**	**3.32 (1.63–6.79)**
Upper extremity	0.61 (0.14–2.77)	0.78 (0.14–4.34)
Lower extremity	**3.43 (1.95–6.01)**	1.05 (0.49–2.27)
LOS (acute care unit at the trauma center)	**1.06 (1.03–1.10)**	**1.12 (1.08–1.16)**

Significant results are presented in bold

Local hospital (*n* = 256) consists of local hospitals (*n* = 237, 92.6%) and nursing home/inpatient community-based rehabilitation (*n* = 19, 7.4%)

*RRR* relative risk ratio, *CI* confidence interval, *NCI* Norwegian Centrality Index, *ASA* American Society of Anesthesiologists Classification, *NISS* New Injury Severity Score, *AIS* Abbreviated Injury Scale, *LOS* length of hospital stay

Table 4 presents the findings of the multivariable multinomial logistic regression analysis, which revealed that both sociodemographic and injury-related factors had a significant influence on the RRR for discharge to specialized rehabilitation and a local hospital compared with discharge home. Children (< 18 years) had a significantly lower RRR for discharge to a local hospital compared with the 35–64-year age group. NCI 3–4 significantly increased the RRR for discharge to a local hospital (RRR 3.61, 95% CI 2.07–6.27) compared with NCI 1–2. Higher ASA led to a significantly higher RRR for discharge to a local hospital. An increased NISS and number of injuries increased the RRR for discharge to both local hospital and specialized rehabilitation compared with home, whereas an increase in the LOS increased the RRR for discharge to specialized rehabilitation compared with home. Patients assigned AIS ≥ 3 for head injury (RRR 6.1, 95% CI 2.80–13.38) or spinal injury (RRR 8.2, 95% CI 3.13–21.30) had a significantly increased RRR for discharge to specialized rehabilitation compared with patients with AIS < 3 injuries. Patients with AIS ≥ 3 spinal or lower extremity injuries had a significantly higher RRR for discharge to a local hospital rather than home compared with patients with AIS < 3 spinal or lower extremity injuries. However, having an abdominal injury with AIS ≥ 3 significantly decreased the RRR for discharge to specialized rehabilitation and local hospitals. The model had a McFadden *r*^2^ of 0.31 indicating that the overall multivariable multinomial logistic regression model performance is acceptable. An additional sensitivity analysis where there was adjusted for the trauma center patients belonged to did not change most of the model predictors. However, NCI 5–6 now became a statistically significant predictor for discharge to local hospital, and NCI 3–4 for discharge to specialized rehabilitation. See Table 5 in the Appendix.

**Table 4:** Multivariable multinomial logistic regression analysis for sociodemographic and injury-related characteristics

Characteristic	Full multivariable model, RRRs (95% CI)	
*n* = 601	Local hospital versus Home	Specialized rehabilitation versus Home
Sociodemographic characteristics		
Sex		
Male	1	1
Female	1.36 (0.79–2.33)	1.08 (0.55–2.10)
Age at inclusion (years)		
0–17	**0.32 (0.13–0.78)**	0.72 (0.28–1.87)
18–34	0.77 (0.41–1.43)	1.17 (0.56–2.45)
35–64	1	1
≥ 65	1.71 (0.91–3.21)	1.10 (0.50–2.44)
Centrality Index (NCI)		
Category 1 and 2	1	1
Category 3 and 4	**3.61 (2.07–6.27)**	1.89 (0.98–3.64)
Category 5 and 6	0.98 (0.44–2.20)	0.90 (0.35–2.29)
Preinjury ASA		
1	1	1
2	**2.08 (1.15–3.75)**	1.45 (0.72–2.91)
3 and 4	**2.67 (1.20–5.94)**	0.69 (0.23–2.07)
Injury- and treatment-related characteristics		
Injury mechanism		
Falls	1.30 (0.76–2.23)	1.17 (0.61–2.36)
Transport-related	1	1
Violence	0.17 (0.03–1.03)	0.32 (0.63–1.65)
Others	1.63 (0.82–3.22)	0.71 (0.30–1.71)
NISS		
Moderate (NISS 10–15)	1	1
Severe (NISS > 15)	**2.17 (1.18–4.01)**	**2.93 (1.27–6.75)**
Number of injuries	**1.31 (1.19–1.44)**	**1.23 (1.10–1.37)**
Body regions with AIS ≥ 3		
Head	0.99 (0.52–1.88)	**6.12 (2.80–13.38)**
Face	1.22 (0.22–6.67)	0.33 (0.04–2.59)
Neck	2.27 (0.25–20.68)	5.84 (0.49–69.35)
Thorax	0.80 (0.43–1.49)	0.46 (0.21–1.03)
Abdomen	**0.14 (0.06–0.33)**	**0.14 (0.04–0.44)**
Spine	**4.01 (1.74–9.20)**	**8.16 (3.13–21.30)**
Upper extremity	0.45 (0.06–3.26)	1.23 (0.09–16.07)
Lower extremity	**3.19 (1.52–6.70)**	0.67 (0.23–2.02)
LOS (acute care unit at the trauma center)	1.01 (0.97–1.06)	**1.11 (1.06–1.17)**

Local hospital (*n* = 256) consists of local hospitals (*n* = 237, 92.6%) and nursing home/inpatient community-based rehabilitation (*n* = 19, 7.4%)

Significant results are presented in bold

RRR relative risk ratio, CI confidence interval, NCI Norwegian Centrality Index, ASA American Society of Anesthesiologists Classification, NISS New Injury Severity Score, AIS Abbreviated Injury Scale, LOS length of hospital stay

## Discussion

### Key results

In this prospective, longitudinal, population-based study on patients of all ages from two of the trauma centers in Norway, we demonstrated that sociodemographic and injury-related factors influenced discharge destination after acute care in individuals with moderate-to-severe injuries. In addition, we further described the patients’ sociodemographic factors, including geographical differences based on the NCI.

The results demonstrated that despite most patients (76%) having severe injuries (NISS > 15), only 22% of patients were discharged directly to specialized rehabilitation from the trauma center, 35% were discharged home, and 43% were discharged to a local hospital. Children were primarily discharged home, while patients ≥ 65 years were frequently discharged to their local hospital. Increased severity of head and spinal injuries was associated with discharge to specialized rehabilitation, whereas increased severity of injury in the lower extremities was associated with discharge to a local hospital rather than home. In addition, living in less central regions (NCI 3–4) was associated with discharge to a local hospital than home. The head and thorax were the body regions most often injured overall and among injuries with AIS ≥ 3. The proportion of patients with severe injuries was highest for the group of patients assigned NCI 3–4, and falls were less prominent as a cause of injury for patients assigned NCI 5–6. Furthermore, these patients had longer LOS than patients living in more central areas.

### Discharge destination

In line with our hypotheses, children had a lower RRR for discharge to a local hospital versus home, using the discharge destination of patients in the 35–64-year age group as a reference. Chen et al. reported similar results (reference group: 35–44 years); however, their study comprised only patients with TBI (Chen et al. 2012). In addition, they found that patients under the age of 18 years were significantly less likely to be discharged to inpatient rehabilitation (odds ratio [OR] 0.09), a result that was not demonstrated in our study. In an observational study based on data from 2004 to 2013, Nesje et al. (2019) found that the majority of children received by a trauma team at a Norwegian trauma center was discharged home. Similar results were reported in a recent study by Dahl et al. (2021) that assessed the epidemiological characteristics of children with TBI. These findings reflect that the children may have caregivers (e.g., parents) who can take care of them at home, and it may be preferred for children to receive their rehabilitation at home. Another contributing factor is probably that neuro rehabilitation for children in Norway is less developed than for the adult population, especially for children under 6 years of age (Dahl et al. 2021). Furthermore, it is likely that children have certain patterns of injury, such as a lower frequency of orthopedic injuries in the lower limbs.

Elderly trauma patients (defined as ≥ 65 years in the cited study) are at an increased risk of morbidity and mortality after injury (Kocuvan et al. 2016). Determining the best discharge destination for patients in this population may be difficult as this decision is based on the medical, functional, and social aspects of the patient’s injury in association with their preinjury medical status (Shepperd et al. 2010) and the availability of rehabilitation (Sveen et al. 2016). The proportion of patients discharged to rehabilitation declines as the ASA score increases, which is in line with a recently published study on factors associated with the direct pathway to specialized rehabilitation after TBI in Norway (Tverdal et al. 2021). Higher ASA leads to a significantly higher likelihood of discharge to a local hospital; this is in line with Chen et al.’s study on patients with TBI, where they found that an increase in the Charlson Comorbidity Index increased the OR for discharge to a local hospital (Chen et al. 2012). Our results also reflect the findings of a study by Beaulieu et al. on trauma patients, where patients discharged to non-home locations were older than those discharged to rehabilitation, who in turn were older than those who were discharged home (Beaulieu et al. 2014). In our study, we did not find that age was associated with discharge destination for adult patients given the three chosen age categories. This contrasts with the findings of a Norwegian TBI study, where young adults were discharged directly to specialized rehabilitation more often than older patients (Sveen et al. 2016). In our multivariable analysis, we found no significant association between ASA group and discharge to specialized rehabilitation compared to discharge to home. Regarding substance dependence, our results are in line with those of Tverdal et al. (2021) who found no support for the notion that patients with preinjury substance dependence were downgraded from the direct pathway to specialized rehabilitation following moderate-to-severe TBI. This findings are also in line with Beaulieu et al.’s (2014) report of no relationship between having a psychiatric comorbidity and discharge destination in trauma patients.

As expected, and in line with previous studies, we found that the probability of discharge to specialized rehabilitation and local hospital compared to home significantly increased with more severe trauma and an increased number of injuries. These results corroborate evidence from previous studies (Debus et al. 2016; Zarshenas et al. 2019). Furthermore, there was a positive association between LOS and discharge to specialized rehabilitation. A study exploring factors predicting discharge destination after a fall with fracture at any body region found that higher ISS and longer LOS in the ICU increased the odds for discharge to inpatient rehabilitation or a skilled nursing facility compared with home (James et al. 2018). In addition, Beaulieu et al. found that patients discharged to rehabilitation facilities had a higher mean ISS and LOS compared with patients who were discharged home (Beaulieu et al. 2014). Furthermore, Chen et al. reported significantly increased odds for discharge to rehabilitation for patients with LOS ≥ 25 days compared with patients with LOS < 25 days in a cohort of patients with TBI (Chen et al. 2012). In another study exploring predictors of discharge destination in patients with major traumatic injury, Khorgami et al. found that an ICU LOS longer than 5 days, ISS > 15, or specific injuries (lower extremity fracture, pelvic fracture, intracranial hemorrhage, spinal fracture) could predict the need for discharge to a facility (Khorgami et al. 2019).

Patients with AIS ≥ 3 for head or spinal injury were significantly more likely to be discharged to specialized rehabilitation or local hospitals than patients with AIS < 3 head or spinal injuries. This result was expected and is consistent with the findings of Debus et al., who showed that AIS pelvis ≥ 2, AIS legs ≥ 2, AIS spine ≥ 4, and AIS head ≥ 3 were independent factors associated with discharge to a rehabilitation clinic (Debus et al. 2016). In our study, patients with AIS ≥ 3 lower extremity injuries were significantly more likely to be discharged to a local hospital than home compared with patients with AIS < 3 lower extremity injuries, which is in line with the study by Khorgami et al. (2019). It is conceivable that patients with lower limb injuries need support in activities of daily living before they regain independence in these functions.

A contrasting result was that the group of patients who sustained severe abdominal injuries (AIS ≥ 3) were significantly less likely to be discharged to specialized rehabilitation or local hospitals than home compared with the group of patients with less severe abdominal injuries. A possible explanation is that many patients with injuries to the liver, spleen, or kidney who do not have other injuries will be treated without surgery and will be discharged home after a few days in the trauma center. Those who require operative treatment (i.e., laparotomy) but did not sustain other severe injuries will be discharged home when their bowel function is re-established.

Comparing the discharge destination between the two centers (UNN and OUH) revealed a higher proportion discharged home and to specialized rehabilitation at UNN. The results must be interpreted with caution, as there are few patients at UNN. A possible reason could be local guidelines and traditions, or capacity. To guide discharge to specialized rehabilitation, The Norwegian Trauma Care Guidelines from 2017 should apply, as they provide recommendations for acute rehabilitation after severe trauma (Wisborg et al. 2017). The routines are also guided by the National Institute for Health and Care Excellence that patients with an ISS > 9 in a major trauma center or trauma unit should be assessed for rehabilitation needs, and a rehabilitation prescription should be provided for all patients deemed to have those rehabilitation needs (NICE guideline [NG40] 2016). However, practical guidelines will have a pragmatic approach and will probably not prioritize older patients with multiple comorbidities. A study on adherence to these guidelines is ongoing.

### Centrality

The comparison between patients living in areas with different NCIs revealed that a higher proportion of patients residing in NCI 3–4 areas sustained severe injuries. We observed the same result for patients living in NCI 5–6 areas, but to a lesser degree. An important contribution to this result is probably that patients injured in the trauma center’s primary area will be admitted there independent of the severity of their injury as long as the criteria for hospital admission are fulfilled. In contrast, for the patients injured in the trauma center’s secondary area (i.e., patients living in areas assigned a higher NCI), those with the least severe injuries will be admitted to their local hospital, whereas the most severely injured will be sent to the trauma center. OUH and UNN are in NCI 1 and 3 areas respectively. With the reasonable assumption that most patients are injured in the area in which they reside, the patients injured in the trauma center’s primary area will in general have a lower NCI than the patients injured in the secondary area. Heathcote et al. concluded in a recent study that compared to major cities, injuries occurring in rural areas of Australia often involve different mechanisms and result in different types of severe injury (Heathcote et al. 2022). Injuries occurring outside peoples’ homes and traffic-related injuries ‘off road’ were more likely (Heathcote et al. 2022). This could indicate a higher proportion of high-energy traumas and more severe injuries in less central areas. Furthermore, the less central the area, the higher is probably the likelihood of agricultural injuries. Unfortunately, we do not have knowledge of the relationship between NCI and the degree of agriculture. In our study there was a higher proportion of “other” as injury mechanism in the group of patients residing in less central regions, and this could reflect a higher proportion of agricultural injuries. Such injuries could contribute to the higher proportion of severe injured from the less central areas.

Other studies have focused on low energy trauma, and Bakke et al. demonstrated that in a rural area, low energy trauma accounted for 43% of total trauma deaths during a 10-years period, and that it primarily affected the population above 75 years of age (Bakke et al. 2014). Counties in Norway with a more rural settlement pattern have an older population (Statistics Norway 2017), and age could be a confounder in this unadjusted analysis.

Our results revealed that residing in an NCI 3–4 area significantly increased the RRR of being discharged to a local hospital (RRR 3.61, 95% CI 2.07–6.27) compared with residing in an NCI 1–2 area. This observation likely reflects that the trauma centers have two populations of trauma patients: (1) local patients who are admitted to the hospital as a local trauma hospital and (2) patients who are admitted with the regional function. These patients will have a different distribution of NCIs, as a higher proportion of them will be admitted from less central areas. A substantial proportion of the patients residing in Oslo (an NCI 1 area) have OUS as their general local hospital, and will generally not be transferred to another local hospital as a part of the trauma care. A similar effect will be seen for patients in Tromsø and surroundings, as they have UNN as their local hospital, but as this is an NCI 3 area, the effect will have an opposite effect on the observed result.

Some differences in injury patterns were found; patients assigned NCI 1–2 had a lower proportion of AIS ≥ 3 abdominal injuries, and the frequency of severe spinal injury was higher for patients assigned NCI 5–6. The latter is in line with Heathcote et al.’s study, where they found that patients injured in rural regions were more likely to have spinal cord injuries compared to patients injured in major cities (Heathcote et al. 2022). We found that LOS was longer for patients assigned higher NCIs. This might be due to the prevalence of more severe injuries in these areas, which is in line with our pre-study assumption. A scoping review of geographical location and outcomes after trauma reported that most of the included studies reported no difference in mortality between the rural and urban patient groups for those who survived transport to the hospital (Keeves et al. 2019). No consistent trends were identified in the few included studies that reported recovery outcomes (Keeves et al. 2019). The LOS reports were inconsistent among the studies and may reflect the variation in the methodologies used. However, a longer LOS was reported for rural patients in the studies that involved major trauma (ISS > 15) and patients with TBI, which is in line with our study (Keeves et al. 2019).

The identification of factors associated with discharge destination after acute care for patients with moderate-to-severe traumatic injuries can be important from a planning perspective. By providing new data on patients of all ages with all types of moderate-to-severe traumatic injuries and due to its prospective multicenter design covering more than 60% of the trauma center population of Norway, this study can contribute to addressing a knowledge gap that may have hindered stakeholders and policy makers in a proper health care planning.

With the overarching aim of optimizing outcomes for the individual patient, an important next step would be to describe the rehabilitation needs and determine whether the rehabilitation needs are met in this population. This will be a focus for future publications in this project.

### Strengths and limitations

This study was performed during the first wave of the COVID-19 pandemic. It is likely that this significantly influenced the discharge patterns from usual practice. We assume that this effect was most pronounced for specialized rehabilitation, with the possibility of underestimating the proportion of patients discharged under non-pandemic circumstances. However, a study performed at OUH that looked at the period from March 2020 to August 2021 found that for patients with moderate-to-severe TBI, the direct pathway to early specialized rehabilitation was maintained (Tverdal et al. 2022). Dropout analysis (non-responders or declined participation) revealed no significant differences in sex or mean age between the dropouts and the included patients. For the potentially eligible patients with a drug condition, some of them may have discharged themselves against the advice of the attending physician before a two-day hospital stay; therefore, they did not meet this inclusion criterion. This might have introduced bias, as their sociodemographic characteristics, injury- and treatment-related factors, or discharge destinations could have been different from the other patients’. However, we found no significant difference in discharge destination between the included patients with drug conditions and the patients without drug conditions. Previous studies have found that many of individuals with mental disorders do not seek medical help for their condition (Torvik et al. 2018). Thus, the generally high prevalence of undiagnosed mental health and substance use conditions can limit the findings for this variable. The information in the medical records on comorbidities was more complete for patients who had OUH as their local trauma hospital than for the patients admitted to OUH with the regional function, and this could be a source of bias. At UNN, the clinicians have access to a common medical record system for all the hospitals in the health region. Many patients who sustain moderate-to-severe traumatic injuries are admitted to their local trauma hospital without being admitted to a trauma center. If these patients have different profiles than the patients at the trauma centers, this may reduce the generalizability of the data. Furthermore, we cannot rule out some selection bias, such as patients with NISS > 9 who were not admitted to the trauma center, older patients with a high degree of preinjury comorbidity, or patients with non-detected injuries. In addition, patients admitted to the trauma center after more than 72 h were less likely to be identified by us, and a proportion of them thus not registered as “assessed for eligibility”. However, the entire trauma population at the two trauma centers was assessed for inclusion.

A strength of this study is its multicenter design. The population base of the OUH and UNN trauma centers represents over 60% of the Norwegian population, and our findings are likely representative of patients with acute moderate-to-severe injuries admitted to trauma centers on a national level.

Other strengths of this study include its prospective design, the large sample of patients of all ages, the use of the hospitals’ trauma registries to verify the injury severity scores, the small number of eligible patients who were excluded, as well as the small amount of missing data.

## Conclusion

This prospective study included patients of all ages with moderate-to-severe traumatic injuries and revealed that two-thirds of the patients sustained severe traumatic injury and 22% were discharged directly to specialized rehabilitation. The present study further demonstrated that age, centrality of the municipality of residence, preinjury comorbidity, injury severity, LOS, and the number and specific types of injuries were the most important factors influencing discharge destination. The findings can guide stakeholders and policymakers in health care planning.

## Data Availability

The datasets generated and/or analyzed in the current study are not publicly available due to the sensitivity of the material.

## Appendix

See Table 5.

**Table 5:** Multivariable multinomial logistic regression analysis for sociodemographic and injury-related characteristics adjusted for trauma center

Characteristic	Full multivariable model, RRRs (95% CI)	
*n* = 601	Local hospital versus Home	Specialized rehabilitation versus Home
Sociodemographic characteristics		
Sex		
Male	1	1
Female	1.40 (0.81–2.43)	1.10 (0.56–2.17)
Age at inclusion (years)		
0–17	**0.29 (0.12–0.70)**	0.69 (0.27–1.79)
18–34	0.71 (0.38–1.35)	1.16 (0.55–2.42)
35–64	1	1
≥ 65	1.76 (0.93–3.34)	1.13 (0.51–2.52)
Centrality Index (NCI)		
Category 1 and 2	1	1
Category 3 and 4	**4.92 (2.70–8.96)**	**2.29 (1.14–4.59)**
Category 5 and 6	**2.68 (1.03–7.02)**	1.46 (0.47–4.50)
Preinjury ASA		
1	1	1
2	**1.90 (1.04–3.45)**	1.41 (0.70–2.84)
3 and 4	**2.86 (1.27–6.47)**	0.74 (0.25–2.24)
Injury- and treatment-related characteristics		
Injury mechanism		
Falls	1.24 (0.72–2.14)	1.14 (0.60–2.19)
Transport-related	1	1
Violence	0.17 (0.03–1.03)	0.32 (0.06–1.64)
Others	1.52 (0.76–3.05)	0.72 (0.30–1.74)
NISS		
Moderate (NISS 10–15)	1	1
Severe (NISS > 15)	**1.95 (1.04–3.63)**	**2.91 (1.26–6.71)**
Number of injuries	**1.30 (1.18–1.43)**	**1.23 (1.10–1.38)**
Body regions with AIS ≥ 3		
Head	1.03 (0.53–1.98)	**6.04 (2.76–13.25)**
Face	1.94 (0.32–11.70)	0.47 (0.06–3.94)
Neck	1.91 (0.21–17.37)	5.45 (0.47–63.40)
Thorax	0.83 (0.44–1.57)	0.45 (0.20–1.01)
Abdomen	**0.12 (0.05–0.29)**	**0.13 (0.04–0.40)**
Spine	**4.10 (1.76–9.57)**	**8.53 (3.24–22.47)**
Upper extremity	0.38 (0.05–2.94)	1.09 (0.08–14.61)
Lower extremity	**3.54 (1.66–7.55)**	0.69 (0.23–2.08)
LOS (acute care unit at the trauma center)	1.02 (0.98–1.07)	**1.12 (1.07–1.18)**

Local hospital (n = 256) consists of local hospitals (n = 237, 92.6%) and nursing home/inpatient community-based rehabilitation (n = 19, 7.4%)

Significant results are presented in bold

*RRR* relative risk ratio, *CI* confidence interval, *NCI* Norwegian Centrality Index, *ASA* American Society of Anesthesiologists Classification, *NISS* New Injury Severity Score, *AIS* Abbreviated Injury Scale, *LOS* length of hospital stay

## Abbreviations

AIS: Abbreviated injury scale
ANOVA: Analysis of variance
ASA: American Society of Anesthesiologists
CI: Confidence interval
ICU: Intensive care unit
IQR: Interquartile range
ISS: Injury Severity Score
LOS: Length of hospital stay
NCI: Norwegian centrality index
NISS: New Injury Severity Score
OR: Odds ratio
OUH: Oslo University Hospital
RRR: Relative risk ratio
SD: Standard deviation
TBI: Traumatic brain injury
UNN: University Hospital of North Norway
